# Multilevel Analysis of Socioeconomic Determinants on Diabetes Prevalence, Awareness, Treatment and Self-Management in Ethnic Minorities of Yunnan Province, China

**DOI:** 10.3390/ijerph13080751

**Published:** 2016-07-25

**Authors:** Rong Su, Le Cai, Wenlong Cui, Jianhui He, Dingyun You, Allison Golden

**Affiliations:** 1 School of Public Health, Kunming Medical University, 1168 Yu Hua Street, Chun Rong Road, Cheng Gong New City, Kunming 650500, China; surong.endure@163.com (R.S.); cuiwenlong972@126.com (W.C.); hejianhui9804012@163.com (J.H.); youdinyun_km@163.com (D.Y.); agoldena@outlook.com (A.G.); 2 Department of Gerontology, The Affiliated Ganmei Hospital of Kunming Medical University, 504 Qing Nian Road, Kunming 650011, China

**Keywords:** unique ethnic minority, diabetes, prevalence, self-management, multilevel modeling, socioeconomic status

## Abstract

*Objectives*: The objective of this manuscript is to investigate socioeconomic differences in prevalence, awareness, treatment and self-management of diabetes among ethnic minority groups in Yunnan Province, China. *Methods*: We conducted a cross-sectional survey in a sample of 5532 Na Xi, Li Su, Dai and Jing Po ethnic minorities. Multilevel modeling was used to estimate odds ratios (OR) and 95% confidence intervals (CI) for diabetes prevalence, as well as the other outcomes. *Results*: Higher individual educational level was associated with a higher rate of awareness, treatment, adherence to medicines and monitoring of blood glucose (OR = 1.87, 4.89, 4.83, 6.45; 95% CI: 1.26–2.77, 1.87–12.7, 1.95–11.9, 2.23–18.6, respectively). Diabetic respondents with better household assets tended to receive more treatment (OR = 2.81, 95% CI: 1.11–7.12) and to monitor their blood glucose (OR = 3.29, 95% CI: 1.48–7.30). Diabetic patients with better access to medical services were more likely to treat (OR = 7.09, 95% CI: 2.46–20.4) and adhere to medication (OR = 4.14, 95% CI: 1.46–11.7). Income at the contextual level was significantly correlated with diabetes prevalence, treatment and blood glucose monitoring (OR = 1.84, 3.04, 4.34; 95% CI: 1.20–2.83, 1.20–7.73, 1.45–13.0, respectively). *Conclusions*: Future diabetes prevention and intervention programs should take both individual and township-level socioeconomic factors into account in the study regions.

## 1. Introduction

Diabetes has become one of the most common chronic non-communicable diseases in both developed and developing countries over the past several decades. According to the International Diabetes Federation (IDF), there were 382 million people living with diabetes worldwide in 2013, with that number projected to reach 592 million by 2035 [1]. Due to the large economic burden of diabetes and its adverse impact on health, diabetes has become one of the foremost public health challenges of the 21st century [2].

The epidemiological study of diabetes has been conducted in China since 1980, revealing a trend of rapidly increasing diabetes prevalence (from 0.67% in 1980 to 9.7% in 2008) [3,4]. Recent data from the China Non-Communicable Disease Surveillance Group found that the prevalence of diabetes among those 18 and older is estimated to be 11.6%, with approximately 113.9 million adults with diabetes in 2010 [5]. This implies that China is home to the largest diabetic population in the world. While it is well known that there are racial and ethnic disparities in the prevalence of diabetes [6,7], previous research in China has been mainly limited to the Han ethnicity, the majority ethnic group in China. Diabetes research on ethnic minority populations, especially the unique ethnic minorities in Yunnan Province, is currently unavailable. 

There is some evidence that diabetes is not only associated with socioeconomic status (SES) at the individual level [8,9], but also SES at the regional level in developed countries [10,11]. A better understanding of the socioeconomic determinants of diabetes is important to guide the development of effective and targeted diabetes prevention and intervention programs that will have a profound impact on the preventable morbidity and mortality of diabetes. However, few studies have simultaneously explored the impact of individual-level and regional-level SES on diabetes in China. Kewei et al. reported the negative association between SES and the prevalence of diabetes in rural southwest China [12]. However, this study did not examine the relationship between SES and the awareness, treatment and self-management of diabetes. Additionally, the participants in their study were largely of the majority Han ethnicity. Another Chinese study analyzed individual SES differences in diabetes, but did not explore the influence of contextual variables [13].

China has a multiethnic population; different ethnic groups have a wide variety of dietary patterns, lifestyles, socioeconomic levels and geographical environments, which lead to different presentations of diabetes. Yunnan Province, an economically-disadvantaged southwest region of China, has the highest diversity of minorities in China. It is home to 52 ethnic groups, accounting for one-third of the province’s population. Na Xi, Li Su, Dai and Jing Po groups represent four of 15 ethnic minorities unique to Yunnan Province and have a population of 0.31, 0.67, 1.22 and 0.13 million, which accounts for approximately 0.67%, 1.45%, 2.66% and 0.27% of the total population in Yunnan Province in 2010, respectively. Compared to Han people, these unique ethnic groups are relatively more economically disadvantaged, lower in overall education level, lacking in medical resources and more inconvenienced by transportation issues.

Studies have shown that the prevalence of risk factors related to diabetes varies by ethnic population. For example, hypertension prevalence is higher in Na Xi, Li Su, Dai and Jing Po ethnic groups than in Han people of Yunnan Province [14], and central obesity is less prevalent in Dai populations than in Han people [15]. Thus, there may be differences in the prevalence of diabetes between these ethnic groups and Han people.

The present study sought to use multilevel models to assess both individual-level and regional-level socioeconomic differences in prevalence, awareness, treatment and self-management of diabetes among unique ethnic minority groups in Yunnan Province, China.

## 2. Methods

### 2.1. Study Design and Population

The present study employed a multi-ethnic, community-based, cross-sectional health interview and examination survey method conducted in ethnic minority areas of Yunnan Province.

A multi-stage stratified random sampling method was employed to select a representative sample of unique ethnic minorities aged 35 years or older in Yunnan Province. Ethnic residents were defined as greater than five years of residence in their current regions. In the first stage of the study, ethnic minority-inhabited regions of Yunnan Province were divided into “valley area”, “barrage area”, “semi-mountainous” and “mountainous area”, based on the geographical characteristics of the regions. From each of these four areas, we selected one ethnic autonomous prefecture or county, for a total of four counties per region. Four representative unique ethnic minorities (Na Xi, Li Su, Dai and Jing Po, respectively) were sampled from the previously selected four ethnic autonomous prefectures or counties. In the second stage, all of the township districts in the four chosen ethnic autonomous prefectures or counties were classified as economically advantaged, average economic status and economically disadvantaged, according to per capita gross domestic product. A township was then randomly selected in each economical level, with 12 townships in total selected. In the third stage, three villages were randomly chosen using probability proportional to size sampling from each selected township. Finally, based on a simple random sampling method, participants aged 35 or older in each chosen village were identified and invited to take part in our survey. A total of 5600 unique ethnic minority residents from 36 villages participated in the study. Of these, 5532 participants completed both the questionnaire survey and fasting plasma glucose test, yielding a response rate of 98.8%.

This study was approved by the Ethics Committee of Kunming Medical University (Project Identification Code: 2012036) and conducted in compliance with the Declaration of Helsinki. Each study participant gave informed consent for inclusion before participation.

### 2.2. Data Collection

Surveys were carried out between August 2013 and August 2014. Data collection was performed face-to-face by trained interviewers. All interviewers received training on the purpose of the study, the use of the screening questionnaire, the standard method of measurement and the study procedures. 

A structured questionnaire was used to obtain participant demographic characteristics, ethnicity, educational level, average household income, diagnosis, treatment and self-management behaviors of diabetes (including diet control, weight control or weight loss, increase in physical exercise, smoking cessation, medication adherence and monitoring of blood glucose). In addition, information on the type of housing, specifically whether the residence had tap water and a toilet, and walking time to the nearest healthcare facility was also collected. At each stage of data collection, a quality control program was carried out to ensure data accuracy and reliability. 

Fasting blood glucose (FBG) levels were measured for all participants using the ACCU-CHEK Performa glucometer (Roche Diagnostics, Mannheim, German) by taking a small drop of blood from a finger onto a strip of paper the morning after at least 10 h of overnight fasting.

### 2.3. Definitions

Diabetes was defined as FBG ≥ 7.0 mmol/L (≥126 mg/dL) or self-reported previous diagnosis of diabetes by a healthcare professional in a hospital above the county level, based on the 1999 World Health Organization diagnostic criteria [16]. Awareness of diabetes was defined as individuals who had prior knowledge that they had diabetes based on previous physician-diagnosis. Treatment of diabetes was defined as participants who used anti-diabetic medications (oral hypoglycemic agents or insulin) within one month prior to the survey. Control of diabetes was defined as diabetic participants with FBG < 7.0 mmol/L (<126 mg/dL). Medication adherence was defined as diabetic participants taking anti-diabetic drugs prescribed by a doctor in the two weeks prior to the survey. Individuals with diabetes were considered to be taking measures to lower glucose if they reported one of the following behaviors: diet control, weight control or weight loss, increase in physical exercise and/or smoking cessation. Blood sugar monitoring was defined as checking blood glucose levels at least once per month.

Poor household assets were defined as individuals with average annual household income of less than 5,000 Yuan (equal to $805.8 U.S. dollars) and a house made from adobe or stone without tap water or a toilet. Better household assets were defined as individuals with an average annual household income of more than 5,000 Yuan and a house built from wood or brick with tap water and a toilet. Poor accessibility of medical services was defined as individuals with a walking time of more than 30 min from their residence to the nearest healthcare facility, while better accessibility of medical services was defined as less than a 30-min walking time from the residence to the nearest healthcare facility.

### 2.4. Outcome and Independent Variables

The outcome variables included binary measures of diabetes prevalence, awareness, treatment, control and self-management behaviors (taking measures to lower glucose, medication adherence, monitoring blood glucose). Independent variables included demographic indicators (age, gender and ethnicity), socioeconomic status at the individual level (measured by educational level, household assets and accessibility of medical services) and township-level variables, which were assessed by mean yearly income and the percent of residents who had completed primary (Grades 1–6) education or higher in the townships. The socioeconomic characteristics of townships were obtained from the local statistics office.

### 2.5. Statistical Analysis

Data were expressed as numbers or percentages for categorical variables and analyzed using the chi-square test and the chi-square test for trends. Multilevel logistic regression was used to analyze the association between independent variables (age, gender, ethnicity, individual SES variables and township SES variables) and binary individual outcomes, such as prevalence, awareness, treatment of diabetes and taking measures to lower glucose, adherence to medication and monitoring of blood glucose. Individual variables were set at the first level and township variables at the second. The levels of association between independent variables and prevalence, awareness, treatment and self-management behaviors of diabetes were expressed as odds ratios (OR), and we calculated their 95% confidence intervals (CI). R 3.2.0 (The R Foundation for Statistical Computing, Vienna, Austria) was used for all statistical analyses. Statistical significance was based on two-tailed *p*-values, and a value of *p* < 0.05 was considered statistically significant.

## 3. Results

### 3.1. Characteristics of the Study Participants

The participants’ general characteristics are summarized in Table 1. A total of 5532 individuals aged ≥35 years were chosen through the sampling process, including 2677 men and 2855 women. The percent of Na Xi, Li Su, Dai and Jing Po ethnic minorities was 25.3%, 24.7%, 25.3% and 24.7%, respectively. Thirty-point-nine percent of participants had less than a primary education, while 69.1% attended primary school through Grade 6 or higher. Those with poor household assets and poor accessibility of medical services accounted for 70.7% and 33.2% of our study, respectively.

### 3.2. Prevalence, Awareness, Treatment, Control and Self-Management Behaviors of Diabetes in the Study Population

As shown in Table 2, the overall prevalence, awareness, treatment and control rate of diabetes was 4.7%, 63.7%, 48.1% and 24.8%, respectively. Among participants with diabetes reporting a prior diagnosis of diabetes, the rate of taking measures to lower glucose, medication adherence and monitoring of blood glucose was 91.0%, 68.9% and 36.5%, respectively.

The rate of prevalence, awareness and treatment of diabetes significantly increased with increasing age (all *p* < 0.001). Among these four unique ethnic groups, the prevalence of diabetes was the highest in Dai ethnic minorities and the lowest in Li Su ethnic minorities (8.3% vs. 2.3%, *p* < 0.001). The highest rate of awareness was observed in Li Su ethnic minorities; the lowest was observed in Jing Po ethnic minorities (75% vs. 44.6%, *p* = 0.008). Dai ethnic minorities had the highest rate of treatment; Jing Po ethnic minorities had the lowest treatment rate (56% vs. 32.1%, *p* = 0.013). With regard to self-management behaviors of diabetes, the highest rate of medication adherence was found in Dai, and the lowest in Li Su ethnic minorities (82.1% vs. 41.7%, *p* = 0.001). The rate of monitoring blood glucose was highest in Na Xi ethnic minorities; it was the lowest in Li Su ethnic minorities (55% vs. 12.5%, *p* = 0.002). The rate of control and of taking measures to lower glucose did not differ by ethnicity (both *p* > 0.05). There was no significant difference in prevalence, awareness, treatment, control and self-management behaviors of diabetes between men and women among the four unique ethnic minorities (all *p* > 0.05).

### 3.3. Distribution of SES at the Township Level

Table 3 summarizes township contextual variables. Overall, variation in the percent of the population that received a primary education or higher among the 12 townships was high (ranging from 58%–92%).

### 3.4. Multilevel Analysis for the Prevalence, Awareness, Treatment and Self-Management of Diabetes

Table 4 shows multilevel analysis for the prevalence, awareness, treatment and self-management of diabetes. Older participants had a relatively higher prevalence and awareness of diabetes rate (OR = 1.04, 95% CI: 1.02–1.05; and OR = 1.05, 95% CI: 1.04–1.07, respectively).

Individual SES variables in the multilevel modeling showed that more highly-educated participants were more likely to be aware of their condition (OR = 1.87, 95% CI: 1.26–2.77). Diabetic respondents with higher education levels, better household assets and better access to medical services also had a relatively higher treatment rate compared to their counterparts (OR = 4.89, 95% CI: 1.87–12.7; OR = 2.81, 95% CI: 1.11–7.12; and OR = 7.09, 95% CI: 2.46–20.4, respectively). Compared to those with less than a primary school level education, diabetic patients with higher education levels were more likely to adhere to medication and to monitor their blood glucose levels (OR = 4.83, 95% CI: 1.95–11.9; and OR = 6.45, 95% CI: 2.23–18.6, respectively). Among patients with diabetes, compliance to prescribed medicines was more common among those with better accessibility of medical services (OR = 4.14, 95% CI: 1.46–11.7). Diabetic patients with better household assets had an increased likelihood of monitoring their blood glucose levels (OR = 3.29, 95% CI: 1.48–7.30). Ethnicity was found not to be significantly associated with the prevalence, awareness, treatment and self-management behaviors of diabetes.

Among township-level variables, mean yearly income was significantly associated with the rate of prevalence, treatment and monitoring of blood glucose (OR = 1.84, 95% CI: 1.20–2.83; OR = 3.04, 95% CI: 1.20–7.73; and OR = 4.34, 95% CI: 1.45–13.0, respectively). In other words, residents who lived in townships with higher mean yearly income were more likely to suffer from diabetes, receive treatment for the disease and monitor their blood glucose levels after being diagnosed with diabetes.

## 4. Discussion

In the present study, the overall prevalence of diabetes among four unique ethnic groups in Yunnan Province was found to be lower than the national average in 2010 [5], as well as lower than other ethnic populations in China, such as Kazakh (5.9%) [17], She (6.1%) [18], Manchu (8.39%) and Korean Chinese (9.42%) [7]. This disparity in prevalence could be explained by differences in dietary habits, lifestyle, income level and genetic backgrounds. The overall awareness, treatment and control of diabetes in study participants was higher than the previously reported rates in Yunnan Province [13], Liaoning Province [19] and the Uygur and Kazakh populations in Xinjiang [20], but lower than that observed in northeast China [21] and Western countries [22,23]. These findings indicate a need for more effective intervention programs to improve awareness, treatment and control among diabetic patients.

Ethnic differences were significant in regards to prevalence, awareness, treatment and self-management of diabetes in the present study. Among the four unique ethnic groups, diabetes was the most prevalent in Dai ethnic minorities. The lowest rate of awareness and treatment was observed in Jing Po ethnic minorities. Li Su ethnic minorities had the poorest self-management behaviors, including medication adherence and blood sugar monitoring. These results suggest that strategies for improving diabetes prevention and control potentially differ by ethnicity. 

Our findings on the self-management of diabetes among the four different ethnic diabetic respondents revealed that self-management behaviors relating to taking measures to lower glucose were relatively good, while monitoring blood glucose and drug adherence were poor. This indicates that blood sugar monitoring and medication adherence are important aspects to focus on in improving self-management behaviors among the study population in the future. 

Previous studies in developed countries have shown evidence of an inverse relationship between individual-level SES and diabetes: individuals with lower incomes [8,24], lower educational levels [8,9,25] and/or lower occupational classes [26] tend to be more likely to be diabetic. Another study reported that the association between individual SES and diabetes has reversed over time from higher prevalence in the higher SES groups to lower SES ones in low- and middle-income countries [27]. However, significant difference in the association between any individual-level SES and diabetes was not observed in our study. At the community level, we found that diabetes was more prevalent among ethnic residents living in higher-income townships, contrary to the results from studies conducted in developed countries showing higher diabetes prevalence in disadvantaged areas than in affluent ones [10,11]. The reasons for this discrepancy deserve further research. 

Results from previous research show that diabetic patients with a lower level of education [23] or lower household income [13] are less likely to receive treatment. In our study, in addition to individual socioeconomic factors, including education level, household assets and the accessibility of medical services, higher township income levels were also significantly associated with a higher rate of treatment. These findings suggest that greater attention should be given to diabetic patients with lower SES, as well as those living in lower-income regions to achieve a higher rate of treatment. 

A previous study of an ethnically-diverse sample reported that rates of medication adherence were not associated with individual income level [28]. Similarly, no significant relationship between household assets and medication adherence was observed in the present study. However, we found that diabetic respondents with higher individual educational levels and/or better access to medical services were more likely to adhere to medication. Additionally, our findings showed that those in communities with higher income levels had an increased likelihood of compliance to prescribed medicines, which is consistent with the results from randomized controlled trials conducted in the United States [29]. 

Prior studies have shown monitoring of blood sugar to be more common among individuals with higher SES [30]. Our study showed that diabetic individuals with better household assets and those who lived in townships with higher income levels were more likely to monitor their blood glucose, indicating that favorable economic position plays an important role in blood glucose testing. Thus, efforts to promote the development of the economy may have a positive influence on blood glucose monitoring among diabetic patients in the study areas. 

To our knowledge, this study was the first to address prevalence, awareness, treatment, control and self-management of diabetes among unique ethnic groups in China. Moreover, we investigated the association of contextual factors and diabetes using multilevel modeling, a tool that can analyze the influences of individual and contextual SES on health [31]. This approach is rarely used to examine regional effects on diabetes in China. However, there are several limitations in the present study. First, due to the cross-sectional nature of the survey, it is both unable to infer causal relationships or to assess possible trends in socioeconomic differences in diabetes prevalence and other outcomes. Second, the diagnosis of diabetes was based on fasting capillary blood glucose (FCG) tests rather than venous plasma glucose (FPG), which could lead to an underestimation of the prevalence of diabetes. However, prior research demonstrates that the results of FCG and FPG measurements have high consistency, and the FCG test has been shown to be a suitable screening strategy for diabetes [32]. In addition, the requirement that participants fasted for 10 h before the glucose test was an issue for some participants. Thus, in order to ensure that we obtained accurate FBG levels, participants who reported that they failed to fast were asked to get an additional FBG detection on another day. Third, because illiteracy and low educational status are common in our study population, educational level, a marker of SES, was dichotomized into less than primary school education and primary education through Grades 6 or higher, which offers a limited picture of education level. Fourth, some data, including individual socioeconomic indictors and self-care behaviors, were self-reported and, thus, subject to recall biases. Finally, our study did not distinguish between type 1 and type 2 diabetes mellitus. However, as all participants were 35 years of age or older in the present study and type 2 diabetes mellitus is the primary type of diabetes afflicting adults, this study mainly relates to type 2 diabetes mellitus.

## 5. Conclusions

In summary, our multilevel analyses suggest that higher individual educational levels are associated with a higher rate of awareness, treatment, adherence to medicines and monitoring of blood glucose. Diabetic respondents with better household assets are more likely to receive treatment and to monitor their blood glucose. Diabetic patients with better access to medical services are more likely to treat their diabetes and to adhere to prescribed medicines. Income at the contextual level is correlated with diabetes prevalence, treatment and blood glucose monitoring among the four unique ethnic groups. The findings of this study suggest that effective diabetes prevention and intervention strategies need to take into account not only individual SES, but also contextual socioeconomic factors in the study regions.

## Figures and Tables

**Table 1:** General characteristics of the study participants.

Variables	*n*	%
Sex		
Male	2677	48.4
Female	2855	51.6
Age		
35–44 years	1753	31.7
45–54 years	1511	27.3
55–64 years	1238	22.4
≥65 years	1030	18.6
Ethnicity		
Na Xi	1402	25.3
Li Su	1366	24.7
Dai	1397	25.3
Jing Po	1367	24.7
Educational level		
Less than primary education	1708	30.9
Primary education grade 6 or higher	3824	69.1
Household assets		
Poor	3910	70.7
Better	1622	29.3
Accessibility of medical services		
Poor	1834	33.2
Better	3698	66.8
Total	5532	100.0

Notes: Data are presented as numbers or percent.

**Table 2:** Prevalence, awareness, treatment, control and self-management behaviors of diabetes among unique ethnic minorities in southwest China (*n* (%)).

Characteristics	Prevalence	Awareness	Treatment	Control	Self-Management Behaviors of Diabetes		
					Taking Measures to Lower Glucose	Medication Adherence	Monitoring Blood Glucose
Sex							
Male	122 (4.6)	74 (60.7)	55 (45.1)	28 (23.0)	66 (89.2)	50 (67.6)	26 (35.1)
Female	140 (4.9)	93 (66.4)	71 (50.7)	37 (26.4)	86 (92.5)	65 (69.9)	35 (37.6)
Age, years							
35–44	27 (1.5)	9 (33.3)	4 (14.8)	3 (11.1)	6 (66.7)	4 (44.4)	4 (44.4)
45–54	68 (4.5)	38 (55.9)	33 (48.5)	14 (20.6)	37 (97.4)	30 (78.9)	19 (50.0)
55–64	79 (6.4)	57 (72.2)	36 (45.6)	23 (29.1)	53 (93.0)	34 (59.6)	19 (33.3)
≥65	88 (8.5) **^†^**	63 (71.6) **^†^**	53 (60.2) **^†^**	25 (28.4)	56 (88.9)	47 (74.6)	19 (30.2)
Ethnicity							
Na Xi	58 (4.1)	40 (69.0)	31 (53.4)	17 (29.3)	33 (82.5)	24 (60.0)	22 (55.0)
Li Su	32 (2.3)	24 (75.0)	12 (37.5)	8 (25.0)	22 (91.7)	10 (41.7)	3 (12.5)
Dai	116 (8.3)	78 (67.2)	65 (56.0)	33 (28.4)	75 (96.2)	64 (82.1)	31 (39.7)
Jing Po	56 (4.1) **^†^**	25 (44.6) *	18 (32.1) *	7 (12.5)	22 (88.0)	17 (68.0) *	5 (20.0) *
Total	262 (4.7)	167 (63.7)	126 (48.1)	65 (24.8)	152 (91.0)	115 (68.9)	61 (36.5)

Notes: Categorical variables were presented as numbers or percentages and analyzed using the chi-square test or the chi-square test for trends. * Denotes *p* < 0.05 for the difference in multiple comparisons between the four categories of age and ethnicity. **^†^** Denotes *p* < 0.001 for the difference in multiple comparisons between the four categories of age and ethnicity.

**Table 3:** Distribution of socioeconomic status among 12 townships.

Variables	Min.	P_25_	P_50_	P_75_	Max.
Primary (Grades 1–6) education or higher (percent)	58	58	67	75	92
Mean yearly income (Yuan)	2122	2747	3516	4317	4770

Notes: P_25_ denotes the first quartile. P_50_ is the median quartile. P_75_ is the third quartile.

**Table 4:** Multilevel logistic regression analysis of the prevalence, awareness, treatment and self-management of diabetes among unique ethnic minorities in southwest China.

Predictors	Prevalence	Awareness	Treatment	Taking Measures to Lower Glucose	Medication Adherence	Monitoring Blood Glucose
	OR (95% CI)	OR (95% CI)	OR (95% CI)	OR (95% CI)	OR (95% CI)	OR (95% CI)
Individual variables						
Age	1.04 (1.02, 1.05) ^b^	1.05 (1.04, 1.07) ^b^	1.01 (0.96, 1.05)	1.00 (0.95, 1.06)	1.04 (0.99, 1.08)	0.97 (0.93, 1.01)
Gender (reference: male)	1.09 (0.85, 1.40)	1.28 (0.93, 1.78)	1.34 (0.56, 3.20)	1.74 (0.53, 5.68)	1.32 (0.59, 2.97)	1.43 (0.65, 3.17)
Na Xi (reference: Li Su)	1.00 (0.51, 1.95)	1.01 (0.41, 2.52)	1.71 (0.22, 13.2)	4.17 (0.30, 58.9)	0.36 (0.04, 3.38)	2.38 (0.28, 20.0)
Dai (reference: Li Su)	0.86 (0.34, 2.16)	0.30 (0.08, 1.15)	0.26 (0.03, 2.01)	5.79 (0.23, 143)	1.71 (0.24, 12.2)	0.14 (0.01, 1.99)
Jing Po (reference: Li Su)	0.78 (0.34, 1.81)	0.31 (0.09, 1.07)	1.25 (0.17, 9.21)	3.59 (0.19, 68.1)	2.26 (0.33, 15.7)	0.06 (0.00, 0.84)
Educational level (reference: less than primary education)	1.22 (0.90, 1.65)	1.87 (1.26, 2.77) ^a^	4.89 (1.87, 12.7) ^a^	2.42 (0.65, 8.98)	4.83 (1.95, 11.9) ^b^	6.45 (2.23, 18.6) ^b^
Household assets (reference: poor)	1.18 (0.90, 1.54)	1.38 (0.98, 1.95)	2.81 (1.11, 7.12) ^a^	0.38 (0.10, 1.41)	1.38 (0.59, 3.24)	3.29 (1.48, 7.30) ^a^
Medical services accessibility (reference: poor)	0.97 (0.70, 1.34)	0.88 (0.56, 1.36)	7.09 (2.46, 20.4) ^b^	0.99 (0.23, 4.31)	4.14 (1.46, 11.7) ^a^	1.17 (0.39, 3.48)
Township variables						
Percent with primary (Grades 1–6) education or higher	1.01 (0.98, 1.04)	0.99 (0.95, 1.03)	0.93 (0.86, 1.01)	0.99 (0.89, 1.09)	1.02 (0.94, 1.11)	1.00 (0.92, 1.09)
Mean yearly income (×1000 Yuan)	1.84 (1.20, 2.83) ^a^	2.57 (1.21, 5.48)	3.04 (1.20, 7.73) ^a^	1.59 (0.42, 6.00)	1.20 (0.49, 2.93)	4.34 (1.45, 13.0) ^a^

Notes: Table 4 shows the odds ratios (OR) and 95% confidence interval (CI) for the prevalence, awareness, treatment of diabetes and taking measures to lower glucose, medication adherence and monitoring of blood glucose among unique ethnic minorities according to multilevel logistic regression analyses. Individual variables were set at the first level and township variables at the second. A value of *p* < 0.05 was considered statistically significant. ^a^ Denotes *p* < 0.05 for the difference; ^b^ denotes *p* < 0.001 for the difference.
